# A Thesis on Acute Inflammation, Submitted to the Faculty of the Atlanta Medical College, Session 1856

**Published:** 1856-11

**Authors:** C. C. Lloyd

**Affiliations:** Wetumpka, Alabama


A T L A N T A

### Medical and Surgical Journal.

Vol. II.] NOVEMBER, 1856. [No. 3.

### ORIGINAL COMMUNICATIONS.

### ARTICLE I.

*A Thesis on Acute Inflammation, submitted to the Faculty of the Atlanta Medical College, Session* 1856. By C. C. Lloyd, of Wetumpka, Alabama.

*Acute Inflammation.*—The term inflammation is derived from *inflammo*, to burn; so called in consequence of the burning pain felt in the affected part.

The peculiar morbid condition is one of the utmost importance to every surgeon and physician; because there are but few diseases of a serious character which run their course without the occurrence of inflammation at some period of their progress. Hence, the necessity for a most careful investigation, and thorough acquaintance with its phenomena, its diversified products, and the relation of its various processes to each other.

Acute inflammation is that variety which is violent in its attack, and rapid in its course, and may be defined a pathological condition characterized by unusual redness, heat, pain, tumefaction, and change or arrest of the function of the organ or part affected. We may have, however, an occurrence of one, or more, of these symptoms, without the presence of inflammation; nor is the absence of one or more of them incompatible with its existence.

We shall not enter into a detailed consideration of the numerous and diversified causes producing inflammation, but notice them briefly, and proceed to examine its various phenomena, its products, &c. The causes may be divided into predisposing and exciting: of the former, we see examples in those whose vascular systems are irregular, as the result of original conformation, or induced by previous disease—those who have been subjected to fatigue, confinement, impure air, &c. Of the latter, we have mechanical, chemical, and vital irritants; also, those producing congestion—as cold, malaria, suppression of natural or habitual discharges, &c.

The phenomena observed in the process of inflammation are curious and interesting. Place, for example, an irritant, as a drop of nitric acid on a living tissue, and notice the result—the first change visible is a contraction of the vessels, momentary and spasmodic, as it were, retarding the flow of blood through the part. This contraction is speedily followed by great dilatation and increased flow of blood.

The seat of the primary part of the process of inflammation is generally thought to be in the nerves of the part; but Pathologists have not, as yet, proven this satisfactorily; and while this may be true of irritating causes, we are under the impression that the nerves are not essentially its seat; because many of the agencies, producing internal inflammation, seem to produce their effects without any marked implication of the nerves. We are of the opinion, that so far as is at present known, the blood vessels and their contents are the essential seat of the whole process of inflammation; and though irritating causes, as above stated, act on the nerves also, yet others, as cold, operate chiefly on the blood vessels and contents only. Hence, we find that causes predisposing to inflammation, are circumstances interfering with the action of the vascular system.

We next come to notice the changes produced on the vessels and contents, and the attending phenomena. The vessels are enlarged, as is obvious from the redness which may be seen with the naked eye. Enlargement is also seen in congestion; but there are other symptoms which are not seen in congestion. We have greater heat and pain, abundant effusions, a florid redness, instead of lividity, as in congestion, violent pulsation of the arteries, increased motion of the blood, and lastly, the various products which are quite sufficient to satisfy us that we have inflammation.

The causes of, or conditions necessary to, the production of this enlargement of the blood vessels, requires further research. The most plausible theory, however, and the one most generally adopted, is, that the tonicity or irritability of the structure of their walls is impaired, perhaps by the previous excessive stimulation.

Another interesting phenomenon of inflammation is the fact that the flow of the blood is partly increased, and, at the same time, partly diminished. The former condition is seen in the rapid passage of the blood through the arteries; the latter in its stagnation in other obstructed vessels in the part.

Many hypothesis have been framed for the purpose of solving the difficulty which exists in regard to understanding the cause of this obstruction. As far as has been ascertained, it would seem to be due to the increased production of the colorless corpuscles of the blood, and their adhesion to the walls of the vessels, and to each other; to the increased mass of blood in the minute vessels, and to the diminished "*contractilite de tissue*" of their coats.

The blood, also, undergoes important changes. We find the fibrine and white corpuscles greatly increased in this affection. We think the latter, especially, are more in excess, probably, than the fibrine. Now, in estimating the quantity of the latter, the white corpuscles are not taken into the account. They have never yet been separated from the fibrine of the human blood; their composition is unknown; but in the estimate of fibrine, we have their weight included. Now, it is evident, that the white corpuscles are increased, and we think it very probable that a portion of the supposed increase of fibrine may be due to their being weighed with it.

Having noticed the effects of the inflammatory process on the blood vessels and their contents, we will now consider, separately, the symptoms to which we before alluded. The symptoms are divided into local and constitutional. The local occur in the part which is inflamed; the constitutional affect the whole system. The local are, redness, heat, pain, tumefaction, and change in the function of the organ or part.

The redness of an inflamed part, we think to be due chiefly to the increase in the quantity of blood in its vessels, the vessels as before stated are distended, the finest capillaries, which in a normal condition, are invisible, are now distinctly seen, dilated and filled with red blood. *The proportional* increase in red corpuscles, by the exudation of serum, may assist in some degree. Some observers have conceived that new blood vessels are formed by the blood as it forces its way through the tissues. This notion we think erroneous.

*Heat.* The heat of inflammation depends greatly, perhaps solely, on the increased flow of blood through the part. This is evident from the fact that in proportion to the violence of the inflammation is the heat increased. We do not think inflammation, as supposed by some, a calorific process by any means, for many experiments have been instituted to determine this point, and in no instance was the temperature found above that of the interior of the body.

*Tumefaction.* The enlargement of the blood vessels in an inflamed part may assist in producing this phenomenon, but the main agency is the effusion from the distended vessels. The form and degree of the swelling will depend much on the natural structure of the part involved. In the mammary gland, the scrotum or any of those loose cellular structures, the swelling is often extreme, while in parts composed of dense fibrous tissue, but little exists.

*Pain.* The chief cause of pain, is the pressure on the minute nervous branches by the turgid blood vessels: in some degree also, perhaps, by the exalted sensibility of the nerves, which is produced by determination of blood.

This symptom, though usually present in acute inflammation, is not invariably so, neither are we to regard its presence, pathognomonic of this affection. The character and intensity of pain, vary according to the seat of lesion. In inflammation of bone, for instance, or any dense tissue, we have excessive pain, while in parenchymatous, organs and mucous membranes, it is comparatively trifling.

Inflammatory pain is not always confined to the affected part, but seems to radiate along the course of the nerves, manifesting itself in other parts.

Change or arrest of the function of the organ or part, is always present in inflammation. We notice this in gastritis, where the stomach is unable to perform its function of digestion, and in diarrhœa, the morbid secretions poured from the intestines.

*Constitutional symptoms.* Inflammation is attended with certain symptoms called constitutional, the severity of which will be proportional to the intensity of the inflammation, the amount of local irritation, and the vital importance of the organ involved. The constitutional disturbance in inflammation assumes the form of fever. This is called inflammatory, symptomatic or surgical fever. It presents a great variety of forms, the principal of which are called *sthenic* or typical, *asthenic* or typhoid, and *irritative* or nervous.

Inflammation has two true terminations, resolution and metastasis. When it terminates by resolution, we find the effusions removed by absorption, the obstruction which existed in the blood vessels yields, and the part is restored to its normal condition.

By metastasis is meant a change in the seat of the affection, its sudden disappearance in one part and appearance in another. This rarely occurs.

Besides these true terminations of inflammation, we have it passing from its primary condition to other forms of the disease. Thus, where we have effusion of lymph, it is called adhesive; the production of pus, called suppurative; formation of an ulcer, ulcerative; death of the part, gangrenous.

At an early period in the process of inflammation, the turgid blood vessels, in the attempt to relieve themselves, throw out an abundance of serum, resembling that resulting from congestion, except that it contains a little more animal matter. This is followed by the effusion of fibrine, called also lymph, plasma and coagulable lymph, giving to the swelling a degree of hardness as may be seen in boils, thickened mucous, membranes, &c. Of this effused lymph, there are two varieties; called plastic and aplastic, or corpuscular. The plastic is the true coagulable lymph, and plays an important part in the reparation of injuries and rebuilding destroyed tissue. It is met with in the healthy and vigorous. The aplastic or corpuscular, possesses no power of coagulating, but consists chiefly of what are called exudation corpuscles, freely floating in a serous fluid, and is found in cachetic and scrofulous constitions.

The changes which this effused matter may undergo, are numerous, and we will notice them. We find it either absorbed as noticed in resolution, organized or passing into a state of degeneration. In a healthy constitution it is apt to become vascular, and provided the inflammation do not continue too long, and congestion be kept down, will be converted into a fibro-cellular structure. This is called adhesive inflammation. Diversified notions exist among authors, as to the manner in which this vascularization takes place. Are the vessels formed within the lymph? or are they projected into the mass from the adjoining tissue? The latter looks to us the more reasonable, but the subject will admit of further investigation.

As before stated, the effusion may degenerate. When we have a predominance of the aplastic material, found in weak, cachetic or phthisical habits, this is especially apt to occur. This degeneration may take place in various ways. It may become hard and withered; it may undergo what is termed fatty degeneration, and be changed into granular matter, or be converted into pus. This latter is particularly liable to take place when the inflammatory process continues for a considerable length of time, the part is subjected to the influence of the air, or the general health of the patient neglected.

We now approach the suppurative form of inflammation, which is attended with many phenomena of interest. It is characterized by the production of pus. This, when it results from active inflammation, in a healthy subject, is an opaque, creamy, thick fluid, of a yellowish green color, has a slight faint odor, and an alkaline reaction. This is called laudable or healthy pus, and consists essentially of pus-cells, resembling, in some degree, the white corpuscles of the blood. There are several other varieties, the sanious or bloody, the curdy or cheese-like, and the muco-pus or sero-pus.

Pus is, we think, a true product of inflammation, and not the disintegration of the solid tissues, as was thought by the older surgeons.

That form of inflammation which results in the formation of an ulcer, is called ulcerative. The changes produced in the tissues by this process, are not yet clearly understood, but there is no doubt that they consist essentially of the molecular death of the tissues, and the detachment of the dead matter, by a peculiar action of the adjacent living structures.

Lastly, gangrene or sloughing may occur from the intensity of the inflammation. And this may take place, even if the part be sound and the patient's general health unimpaired. Most usually, however, it is where there is debility, local or general, by which the vital power is reduced.

TREATMENT. The treatment may be divided into local and general or constitutional.

In the local treatment, if the inflammation be external, as, for instance, that resulting from a wound, the first indication is to remove all sources of irritation, and as rather a preventative agent, we apply cold evaporating lotions. If the tendency to inflammation be great, the application of cold by irrigation, is very beneficial.

These are the means usually had recourse to in the earliest stages.

When inflammation has actually commenced, the abstraction of the blood locally, by cups or leeches, is found of great benefit. Of course, as the disease progresses, the treatment may be modified, and the surgeon must be guided by the condition of the patient, and the state of the inflamed part.

CONSTITUTIONAL TREATMENT. If the inflammation be but slight, a mild aperient will perhaps fill every indication. But should the sthenic variety present itself, and the patient be strong and plethoric, we must have early recourse to bold and energetic measures. Bloodletting is the most potent and most beneficial remedy we can use. And in the abstraction of blood, we should exercise some precaution as to the position of the patient. He should be placed in a sitting, or better still a standing posture, and be bled from a large orifice, in order to produce the greatest impression upon the system, with the least loss of blood. Cathartics are of very great service. One of the best of these, if not the best, is mercury, or some one of its preparations. Opium is an excellent remedy in this form of symptomatic fever. It exercises a soothing effect upon the nervous system, and also modifies, to some degree, the heart's action. An excellent mode of administering it, is in combination with some one of the mercurials, as calomel.

In the treatment of the asthenic or typhoid variety, we di_ rect our remedies especially to the support of the patient's strength. A gentle laxative may be given to move the bowels, then tonics and stimulants, constitute our most reliable remedies; brandy, wine, ammonia, &c.

In the irritative, the tendency is to great nervous disturbance, sometimes wild raving delirium, followed by extreme exhaustion, and unless relief is had, speedy insensibility and death. Here, we must use anodynes to relieve the violent nervous symptoms, and stimulants to support life's failing powers.

